# Aspects of Dental Occlusion Assessed with the T-Scan System among a Group of Romanian Dental Students in a Cross-Sectional Study

**DOI:** 10.3390/ijerph20064877

**Published:** 2023-03-10

**Authors:** Diana Elena Vlăduțu, Mihaela Ionescu, Lorenzo Noveri, Horia Octavian Manolea, Monica Scrieciu, Sanda Mihaela Popescu, Adina Andreea Turcu, Alexandru Ştefârță, Grigore Lăzărescu, Veronica Mercuț

**Affiliations:** 1 Department of Prosthetic Dentistry, University of Medicine and Pharmacy of Craiova, 200349 Craiova, Romania; 2 Department of Medical Informatics and Biostatistics, University of Medicine and Pharmacy of Craiova, 200349 Craiova, Romania; 3 Studio Dentistico Noveri-Minelli, 18038 Sanremo, Italy; 4 Department of Dental Materials, University of Medicine and Pharmacy of Craiova, 200349 Craiova, Romania; 5 Department of Oral Rehabilitation, University of Medicine and Pharmacy of Craiova, 200349 Craiova, Romania; 6 Department of Orodental Prevention, University of Medicine and Pharmacy of Craiova, 200349 Craiova, Romania

**Keywords:** bruxism, T-Scan, dental occlusion, non-working interferences

## Abstract

This study evaluated the occlusal relationships in students with bruxism, using the T-Scan III system, and their correlation with the activity of the masticatory muscles assessed through surface electromyography (sEMG). The study group was divided into two subgroups (based on self-reporting): 20 participants with possible bruxism and 20 participants without possible bruxism; all participants underwent the following evaluations: sEMG recordings using the dia-BRUXO device for masticatory muscles assessment, as well as static and dynamic occlusion using the T-SCAN III system. The analysis of the maximum intercuspidal (MI) position revealed a positive moderate association between the values of the occlusal forces in MI distributed along the two hemiarches, and the number of grinding events during daytime, which was statistically significant (*p* < 0.05). The analysis of protrusion movements reflected statistically significant differences between the non-working interferences and sEMG parameters specific to bruxism (*p* < 0.05). The analysis of laterotrusion movements indicated that participants with anterolateral guidance presented higher values of awake bruxism indexes and higher values of clenching events during nighttime. For all three mandibular movements, their duration was higher for the study group compared to the control group. Therefore, this study confirmed the utility of sEMG recordings in the bruxism diagnosis process, as well as the association between dental occlusion and bruxism.

## 1. Introduction

Bruxism is an intensely debated condition in the literature due to its clinical and research implications regarding etiology, assessment methods, and clinical manifestations.

Recently, specialists in the field have emphasized the importance of separating SB (sleep bruxism) from AB (awake bruxism), as they could have different etiologies, culminating with the first Consensus on bruxism [1]. In 2012, based on the definitions developed in the 8th edition of the *Glossary of Prosthodontic Terms* (GPT-8) [2], in the second edition of the *International Classification of Sleep Disorders* (ICSD-2) [3], and in the fourth edition of the *Orofacial Pain Guidelines* (OFPG-4) [4], published by the American Academy of Orofacial Pain, a new definition of bruxism was developed that could be used by all specialists. In 2018, the second Consensus on bruxism was developed in which separate definitions were formulated for the two forms of bruxism [5], and later, in 2020, the development of a standardized system for evaluating bruxism was proposed (STAB—Standardized Tool for the Assessment of Bruxism) [6,7,8,9,10,11,12]. According to this standardized system, future research in the field of bruxism will be directed along two axes: Axis A, which will include studies related to the evaluation of bruxism, and Axis B, which will include studies related to the etiology of bruxism, risk factors, and predisposing factors and the importance of the two forms of bruxism (SB and AB) due to different etiologies.

Over time, several etiological theories of bruxism have been formulated. Although these theories are difficult to confirm or disprove due to the controversial nature of the condition, most of them suggest a multifactorial etiology [13,14,15,16,17,18,19,20,21].

Initially, SB was thought to be caused by occlusal factors such as occlusal interferences and premature contacts, with tooth wear confirming the clinical diagnosis.

Currently, in addition to occlusal factors [22], sleep disorders [23,24,25], psycho-behavioral factors [24], and the involvement of central nervous mechanisms are considered [15,17].

The association of bruxism with temporomandibular disorder (TMD) is also studied without being able to specify whether bruxism is a manifestation of TMD or if bruxism causes TMD [26,27,28].

The role of dental occlusion in bruxism was invoked in 1961 by Ramfjord, and subsequent studies supported this concept, as occlusal balancing reduced SB [29,30]. Other studies have disputed the involvement of occlusal imbalances in SB and stated that occlusal therapy does not reduce bruxism [31,32]. Griffin, in 2003, referring to the role of occlusion in bruxism, stated that for the management of bruxism, it is essential to achieve harmony between the centric relation (CR) and maximum intercuspation (MI) [33].

Currently, there are no studies showing that occlusal adjustments (balancing) reduce muscle activity in bruxism, but several studies associate the use of occlusal mouth guard with reduced masticatory muscle activity in bruxism [22,34,35].

Moreover, Manfredini et al. [36] stated, based on a review of the specialized literature, that there is still a lack of methodological studies that definitively reject the importance of occlusal factors in the etiology of bruxism.

Starting from these data, the present study aims to evaluate static and dynamic dental occlusion, using the T-Scan III system to highlight aspects of dental occlusion that may be associated with the presence of bruxism. To establish an association between occlusal factors and bruxism, the results obtained by T-Scan analysis were correlated with those obtained by surface electromyographic (sEMG) recording of the masseter muscle [37]. The null hypothesis would be that there are no associations between occlusal factors and bruxism.

The present research is a cross-sectional study conducted on students from the Faculty of Dental Medicine Craiova, Romania.

This study falls under Axis B according to STAB, namely establishing the role of occlusal factors in the etiology of bruxism.

## 2. Materials and Methods

### 2.1. Participants’ Selection Process

The present research was carried out during the years 2021–2022 within the Faculty of Dental Medicine Craiova on a number of 328 students, aged between 21 and 41 years, of both genders [24,37]. Inclusion in the study was performed on a voluntary basis, and the diagnosis of “possible” and “certain” bruxism was made according to the recommendations of the Consensus on bruxism [5] and the recommendations of STAB [11]. The group formation of participants was limited by the conditions imposed by the COVID-19 pandemic, during which education took place in a hybrid manner.

Initially, based on a questionnaire that included data on the presence of bruxism episodes, the diagnosis of “possible” bruxism was established in 129 students; the difference of 199 students showed no signs of bruxism. From these, 20 students with “possible” bruxism were selected who constituted the study group, and 20 students without signs of bruxism constituted the control group [37].

In order to confirm the diagnosis of “certain” bruxism in the study group and to disprove muscle activity specific to bruxism in the control group, the 40 study participants were subjected to surface electromyographic (EMG) recordings of the masseter muscle using the electromyograph dia-BRUXO for 24 h. Subsequently, all 40 study participants had their occlusal relationships recorded using the T-Scan III system.

The inclusion criteria were based on informed consent for participation in the study, and the exclusion criteria were the presence of edentulous gaps or prosthetic restorations, periodontal diseases, active pathological processes in the dento-maxillary system, the presence of orthodontic appliances, the presence of general conditions that require the administration of anti-inflammatories, sedatives, or muscle relaxants. In addition, on the days when the recordings were made, the evaluated students did not consume alcohol, tobacco, or coffee.

The study was conducted after obtaining informed consent regarding the objectives and method of conducting the study, in compliance with the Declaration of Helsinki. The approval of the Ethics Commission of UMF Craiova (no. 84/03.06.2021) was obtained in advance.

### 2.2. sEMG Recordings

The dia-BRUXO electromyograph is a portable type; it has a single recording channel and is manufactured by Biotechnovations S.R.L., based in Via G. Matteotti, 189-18038 Sanremo IM [8,37,38].

The most important parameters exported by the dia-BRUXO device were: “BTI—Bruxism Time Index” which represents the time interval in which a person has bruxism episodes from the total recorded period, “BWI—Bruxism Effort Index” which represents a percentage between the effort exerted by the masseter muscle during bruxism episodes, compared to the maximum effort that the masseter muscle could exert in the same period of time, and the “BPI—Personal Bruxism Index”, which represents a parameter of the severity of bruxism for a given patient, so that different cases can be ordered according to severity. In addition to these parameters, it provided data describing the type of recorded events: tonic contractions, phasic contractions, mandibular bracing, mandibular thrusting, light grinding, and severe grinding [37].

### 2.3. Occlusal Recordings with the T-Scan System

T-Scan is a digital static and dynamic occlusion analysis system that records and measures the contacts between the teeth of the two arches in terms of occlusal force, dynamics of occlusal ratios, and even the duration of these occlusal contacts (expressed in seconds). The T-Scan III system from Tekscan Inc. (South Boston, MA, USA) was used in this study.

The T-Scan system consists of the Microsoft Windows T-Scan software (version 212243), associated hardware, the “Evolution Handle (EH-2)”, sensor holders, and special Tekscan sensors.

The “Evolution Handle (EH-2)” collects the data from the sensor and sends it to the computer for processing. The sensors are pressure sensitive and are embedded in a flexible, 0.1 mm thin device that is shaped like a dental arch [21].

In this research, the assessment of occlusion reports for each participant included in both the study group and the control group was carried out in the early hours of the morning.

For occlusal recordings, study participants were seated in the dental chair with the head in the spine extension. Participants were explained how the recordings would proceed, and then they were performed.

The occlusion ratios in the MI position (distribution of occlusal forces on the right or left hemiarch and occlusion and disocclusion time), protrusion movement (presence of incisal guidance, working side interferences and non-working side interferences, premature working side contacts and premature non-working (posterior) side contacts in edge-to-edge position) and right and left laterotrusion movement (type of lateral guidance, working-side (laterotrusive) interferences and non-working-side (mediotrusive) interferences, premature contacts of working and non-working side in edge-to-edge position, duration of movement) were evaluated. The data obtained by the occlusal recordings with the T-Scan device were entered into the same Excel table and later correlated with those obtained by sEMG.

### 2.4. Statistical Analysis

Statistical tests were applied using SPSS (Statistical Package for Social Sciences) software, version 20 (SPSS Inc., Chicago, IL, USA). Continuous variables were defined as mean ± standard deviation (SD), while frequency distributions and percentages were used to describe nominal and ordinal variables. Normality distribution was assessed using the Kolmogorov–Smirnov/Shapiro–Wilk test. Correlations and comparisons between the study group and the control group were performed with Kendall’s tau-b, Mann–Whitney U, and Kruskal–Wallis tests for continuous non-normally distributed data and the Chi-Square test for categorical data. All *p*-values less than 0.05 were considered statistically significant.

## 3. Results

### 3.1. Characteristics of the Study Groups

Following the application of the inclusion and exclusion criteria, the study group of 20 participants with “possible bruxism” and the control group of 20 participants without self-reported bruxism presented the following age and gender distributions (Table 1):

All 20 participants in the study group were diagnosed with “certain” bruxism following sEMG recordings.

The sEMG recordings provided the following values for the recorded parameters (Table 2):

### 3.2. Occlusal Recordings

Recorded data are presented in 2D, 3D, or as dynamic movies.

#### 3.2.1. Evaluation of Recordings in the Position of MI

**Figure 1:**
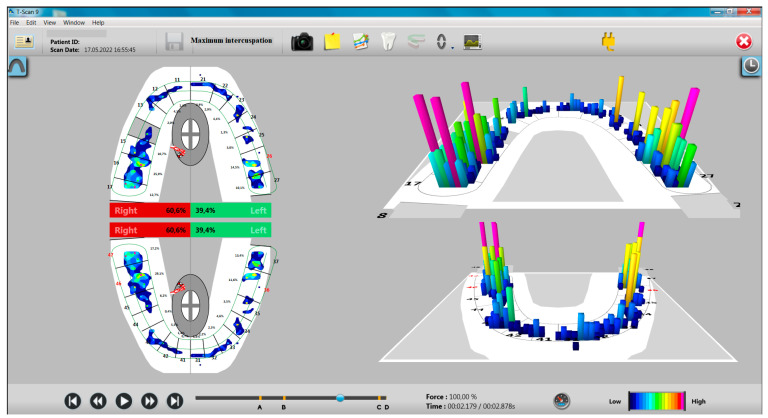
Aspects of T-Scan recording in MI for a participant with bruxism.

**Figure 2:**
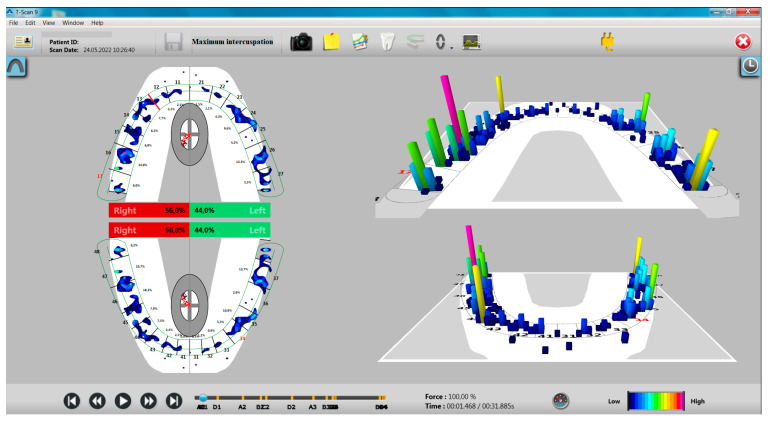
Aspects of T-Scan recording in MI for a participant without bruxism.

The distribution of occlusal forces at the level of the dental arches showed a higher share of occlusal forces at the level of the right hemiarch, especially in the study group with bruxism (Figure 1 and Figure 2).

Similarly, the gender distribution indicated a greater proportion of occlusal forces at the level of the right hemiarch in women, while men presented more balanced values (Table 3).

No statistically significant differences were identified between the study group and the control group or between women and men, regarding the distribution of occlusal forces at the level of the two hemiarches (*p* > 0.05).

For the statistical analysis of the distribution of occlusal forces in the MI between the two hemiarches and the data obtained by sEMG with the dia-BRUXO device, an index of the balance between the occlusal forces at the level of the two hemiarches was calculated, representing the difference of the two values (a higher value of the index represents a greater imbalance between the two occlusal forces). The correlation was analyzed using Kendall’s tau-b test.

A single moderate positive association was identified between the values of the occlusal forces in the MI distributed at the level of the two hemiarches and the number of “grinding” type events during the wakefulness period, which was statistically significant, τ_b_ = 0.226, *p* = 0.042.

Regarding the closing and opening time from the MI position, there are no statistically significant differences between the values recorded in the two groups (Table 4), although the closing/opening times were higher in the study group. There are no statistically significant differences by gender either (Table 4).

The correlation between the closing and opening time from the MI position and the parameters provided by the sEMG recordings with the dia-BRUXO device was analyzed using Kendall’s tau-b test.

A single moderate positive association was identified between the closing time and the number of “other” type events during the wakefulness period, which was statistically significant, τ_b_ = 0.276, *p* = 0.014. No association was identified relative to opening time (*p* > 0.05).

#### 3.2.2. Evaluation of Recordings from Mandibular Movements

##### Evaluation of Protrusion Movement

**Figure 3:**
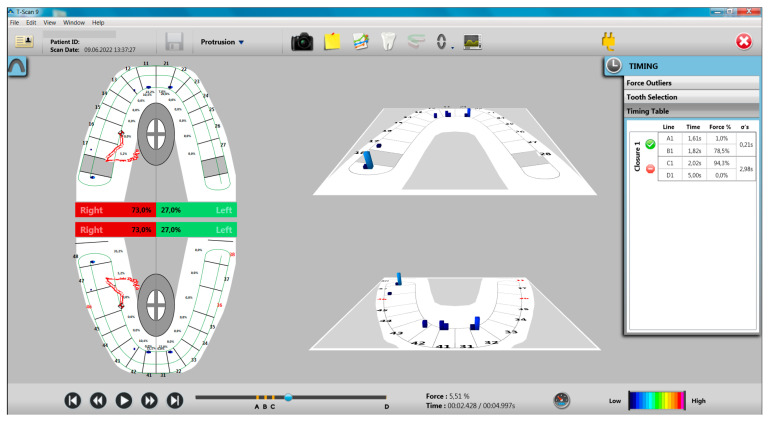
Aspects of the T-Scan recording of the protrusion movement for a participant with bruxism.

The evaluation of the protrusion movement showed that there are no differences between the two groups (study group and control group) in terms of the presence of incisal guidance, and in terms of working and non-working interferences, they were found to be present in higher numbers in the study group compared to the control group, but the differences are not statistically significant (Table 5). At the end of the protrusion movement, there was a reduced number of premature contacts on the working side and on the non-working side (Figure 3).

Associations between the presence of incisal guidance, working/non-working interferences, premature contacts, and parameters provided by sEMG recordings with the dia-BRUXO device were analyzed using the Mann–Whitney U test.

There are no statistically significant correlations between the presence of incisal guidance or the presence of working interferences and sEMG parameters, *p* > 0.05.

Instead, for the groups with and without non-working interferences in the protrusion movement, statistically significant differences were identified relative to several parameters acquired during the night: the SBTI index, the SBPI index, the number of “grinding” type events during the night (Table 6), so participants with non-working interferences had higher values of these parameters, compared to participants without non-working interferences.

The correlation between the duration of the protrusion movement and the parameters provided by the sEMG recordings with the dia-BRUXO device was analyzed using Kendall’s tau-b test.

The following statistically significant associations were identified: SBTI (moderate positive association, τ_b_ = 0.310, *p* = 0.005), SBPI (moderate positive association, τ_b_ = 0.307, *p* = 0.006), percentage of sleep hours (moderate positive association, τ_b_ = 0.288, *p* = 0.009), percentage of waking hours (moderate negative association, τ_b_ = −0.270, *p* = 0.015).

The analysis of the protrusion movement also highlighted the following aspects:

- Participants with incisal guidance showed lower values of occlusal forces distributed on the right hemiarch in MI and higher values of occlusal forces distributed on the left hemiarch in MI, compared to participants without incisal guidance, differences between these groups being statistically significant (*p* < 0.0005);
- Participants with work interferences presented similar values of the distribution of occlusal forces at the level of the right and left hemiarch compared to participants without work interferences;
- Participants with non-working interferences presented higher values of forces distributed on the right hemiarch in MI and lower values of forces distributed on the left hemiarch in MI, compared to participants without non-working interferences, differences between these groups being statistically significant (*p* = 0.027 for forces from the level of the right hemiarch in MI and *p* = 0.025 for the forces from the level of the left hemiarch in MI) (Table 7).

The duration of the protrusion movement had similar values for participants without work interferences (2.39 ± 0.945), and with work interferences (2.36 ± 0.805), so the difference between the two groups was not statistically significant (Table 8). In addition, the duration of the protrusion movement had lower values for participants without non-working interferences (2.31 ± 0.845) compared to participants with non-working interferences (2.45 ± 0.929), and the difference between the two groups was not statistically significant (Table 8).

For a relevant evaluation of the duration of the protrusion movement according to the presence or absence of occlusal interferences, the study group was divided into four categories:

- Group A—participants **without** working interferences and **without** non-working interferences;
- Group B—participants **without** working interferences and **with** non-working interferences;
- Group C—participants **with** working interferences and **without** non-working interferences;
- Group D—participants **with** work interferences and **with** non-work interferences.

The distribution by groups relative to the duration of the protrusion movement and the values of the SBTI, ABTI, SBPI, and ABPI indices is highlighted in Table 9.

The longest mean duration of the protrusion movement was recorded for group B (participants **without** working interferences, but **with** non-working interferences) for participants with AB, followed by group D (participants **with** working interferences and **with** non-working interferences) for participants with SB (Table 9). It is noted that non-working interferences are involved in the increased duration of both movements.

It is worth noting the involvement of occlusal interferences, especially the non-working ones from the protrusion movement, during the mandibular movement, and during the contractions of the masticatory muscles.

##### Evaluation of Laterotrusion Movements

**Figure 4:**
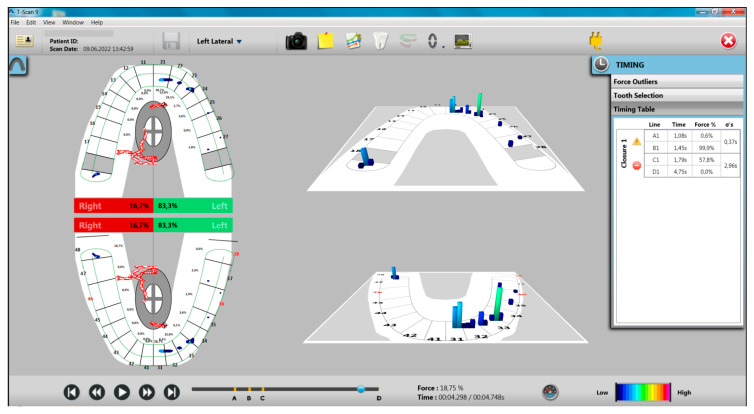
Aspects of T-Scan recording of left laterotrusion movement to a participant with bruxism. Note the antero-lateral group guidance and a non-working interference.

Analyzing the laterotrusion movements, the following aspects were found:

In the left laterotrusion movement from the study group, 50% of the participants presented canine guidance and 50% of the participants presented group guidance (40% antero-lateral, 10% lateral), compared to the control group that presented 90% canine guidance and only 10% showed group guidance (5% antero-lateral guidance and 5% group guidance) (Figure 4).

Clinically, this situation causes a longer disocclusion on the non-working hemiarch, respectively the right hemiarch, a situation also reflected by the recordings made and can generate a higher number of non-working interferences (75%) (Table 10).

Associations between the type of guidance in left laterotrusion movement, working/non-working interferences, premature contacts, and parameters provided by sEMG recordings with the dia-BRUXO device were analyzed using the Kruskal–Wallis H test.

There are no statistically significant correlations between the presence of working interferences or premature working/non-working contacts and the sEMG parameters recorded with the dia-BRUXO system, *p* > 0.05.

Instead, depending on the type of guidance: canine/antero-lateral group/lateral group, statistically significant differences were identified relative to some of the parameters acquired during the day: the ABPI index, the percentage duration of sleep, the percentage duration of the state of wakefulness, the number of “clenching” type events during night, and ABTI (Table 11).

Participants with antero-lateral guidance had higher values for ABTI index, ABPI, percent sleep duration, and number of “clenching” type events during the night.

An important variation between canine-guidance and side-group-guidance participants is the number of “clenching” type events during the night, where the side-group-guidance participants had twice as many events as the canine-guidance group (Table 11).

The correlation between the duration of the left laterotrusion movement and the parameters provided by the sEMG recordings with the dia-BRUXO device was analyzed using Kendall’s tau-b test. The following statistically significant associations were identified: SBTI (moderate positive association, τ_b_ = 0.305, *p* = 0.006), SBWI (moderate positive association, τ_b_ = 0.285, *p* = 0.010), SBPI (moderate positive association, τ_b_ = 0.294, *p* = 0.008), the number of “grinding” type events during the night (moderate positive association, τ_b_ = 0.259, *p* = 0.023), the number of “other” type events during the day (moderate negative association, τ_b_ = −0.297, *p* = 0.008).

**Figure 5:**
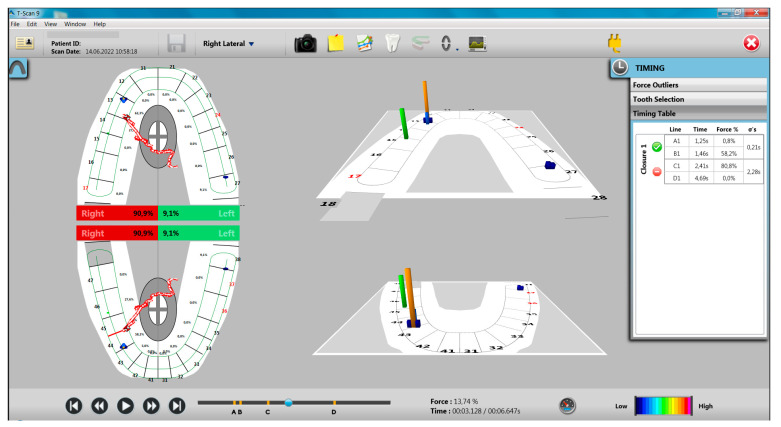
Aspects of T-Scan recording in right laterotrusion movement to a participant with bruxism. Canine guidance and the presence of working side interferences and non-working side interferences are noted.

In the right laterotrusion movement, for the study group, the weight of the group guidance was lower compared to the left laterotrusion movement, respectively, 40% (30% antero-lateral guidance and 10% group guidance), but higher compared to the group control with 25% group guidance (20% antero-lateral guidance and 5% group guidance) compared to the left hemiarch with 10% (Figure 5, Table 12).

Associations between guidance type in right late laterotrusion movement, working/non-working interferences, premature contacts, and parameters provided by sEMG recordings with the dia-BRUXO device were analyzed using the Kruskal–Wallis H test.

No statistically significant correlations were revealed between the presence of working interferences or premature working/non-working contacts and the sEMG parameters recorded with the dia-BRUXO system, *p* > 0.05.

The correlation between the duration of the right laterotrusion movement and the parameters provided by the sEMG recordings with the dia-BRUXO device was analyzed using Kendall’s tau-b test.

The following statistically significant associations of the duration of the right laterotrusion movement were identified: SBTI (moderate positive association, τ_b_ = 0.325, *p* = 0.003), SBWI (moderate positive association, τ_b_ = 0.311, *p* = 0.005), SBPI (moderate positive association, τ_b_ = 0.325, *p* = 0.003), the number of “grinding” type events during the night (moderate positive association, τ_b_ = 0.243, *p* = 0.029), the number of “clenching” type events during the night (positive association moderate, τ_b_ = 0.250, *p* = 0.028), the number of “other” type events during the night (moderate positive association, τ_b_ = 0.354, *p* = 0.005).

Analyzing the duration of the three mandibular movements, it is found that, for all movements, the study group had higher values compared to the control group. The largest difference between the groups was for the protrusion movement (the study group had a mean of 2.60 ± 0.98 s, compared to the control group, with a mean of 2.16 ± 0.71 s).

In the case of laterotrusion movements, higher values were recorded for the study group, especially the laterotrusion movement had a longer duration for the study group with an average of 2.81 ± 0.80 s compared to the control group, with an average of 2.26 ± 0.78 s, the differences in the values recorded in both groups being statistically significant (Table 13).

Correlating the data obtained with the T-Scan device with those obtained by sEMG relative to time, the following aspects were found in the two groups of participants.

Analysis of BTI indices, both daytime and nighttime, revealed that there are moderate positive associations of BTI with the duration of protrusion and laterotrusion movements at night, but not during the day, or with other words, the duration of masticatory muscle contractions in participants with SB correlated with the duration of protrusion and laterotrusion movements (Table 14).

**Figure 6:**
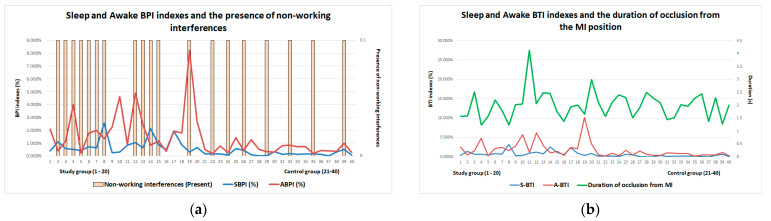
Graphical representation of diaBRUXO and T-Scan indexes: (**a**) Sleep and awake BPI indexes and the presence of occlusal non-working interferences in propulsion; (**b**) sleep and awake BPI indexes and the duration of mandibular movements recorded with the T-Scan system.

According to these results, there are greater changes in dental occlusion in the study participants who were diagnosed with bruxism. However, graphically representing BPI, the most representative index of bruxism, with the presence of occlusal non-working interferences in propulsion (Figure 6a) and BTI, the index that refers to the duration of bruxism episodes, with the duration of mandibular movements recorded with the T-Scan system (Figure 6b), it was found that there is no direct relationship (interdependence) between these parameters, which means that a number of other factors are also involved in bruxism.

## 4. Discussion

The masticatory system is a complex unit designed to perform the basic functions for life (mastication, swallowing, phonation). There is a close interdependence between all the components of the masticatory system so that a disorder in one of its components can induce changes in all components.

The role of occlusal imbalances in bruxism is a critical topic for dental specialists. If occlusal factors are indeed involved in the etiology of bruxism, then the dentist is responsible for providing appropriate therapy, as dentists are the only competent professionals who can improve dental occlusion [39].

The present cross-sectional study aimed to evaluate dental occlusion in a group of students diagnosed with bruxism compared to students without bruxism. The present research was focused, in particular, on highlighting premature contacts and occlusal interferences, which may be associated with bruxism, which is why closing and opening movements from the MI position, mandibular protrusion movements, and right and left laterotrusion movements were evaluated. The duration of closing–opening movements in the MI position as well as the duration of mandibular movements, were also evaluated.

Methods for evaluating occlusal contacts can be divided into qualitative analysis and quantitative analysis.

The qualitative analysis involves evaluating the contacts of the opposing teeth and their positions by means of an occlusal film, respectively, articulation paper, wax, and silicone [40]. The major disadvantage of these recordings is the lack of objectivity and reproducibility, as well as the difficulty in describing the state of occlusion.

The quantitative analysis involves the use of some indices to evaluate the occlusal state. The digital occlusal analysis is one of the best quantitative analysis methods because it measures occlusal forces with high precision and repeatability [41]. Most of the traditional methods analyze static occlusion, but occlusion is not static but rather a parameter of a dynamic structure.

The T-Scan III system developed by Tekscan (Boston, MA, USA) uses the computer to analyze the occlusion in dynamics, allowing both qualitative and quantitative occlusal analyses to be performed simultaneously and with high precision. In fact, during the past years, scientists have actively involved technology through software applications or medical devices to increase the accuracy of the diagnostic process in dentistry and in medicine in general [24,25,26]. The T-Scan system can measure the sequence of dental contacts and quantitative dynamics in relation to time, locate excessive occlusal forces, and record the time from the first contact to the end of the movement [42].

The evolution of T-Scan technology spans the past 40 years and started with T-Scan I, later with the development of Turbo recording in 2008, to the 2014 version known as T-Scan 8 (Tekscan Inc., South Boston, MA, USA) [43]. Sanjana Devi et al. carried out a review of occlusal analysis methods in which they described the evolution of the T-Scan system [44].

Other studies have also been conducted using this method and have highlighted its advantages. Popșor et al. in 2010 and in 2019 [45,46] and Komali G et al. in 2019 [47] showed that the T-Scan is a modern diagnostic tool that can measure relative occlusal forces in real time and is a significant step forward in the modern era of dentistry. It is a reliable indicator of occlusal interferences for their precise identification and occlusal balancing in TMD management, thus contributing to successful treatment. Kalachev et al. also showed that T-Scan is a valuable and reliable system for locating and distributing occlusal contacts in dynamics [48,49].

For bruxism, the T-Scan digital evaluation of occlusion relationships was described by Thanathornwong B and Suebnukarn S in 2017 [41] to show the effectiveness of the method

The results of the present study showed that the participants with bruxism presented greater imbalances at the level of dental occlusion, in terms of the distribution of occlusal forces at the level of the two hemiarches, the presence of non-working interferences in the propulsion movement and in terms of the duration of mandibular movements. Other studies have also addressed this aspect.

Thus, a recent study by Iacob et al. in 2022, by clinical assessment, showed that study participants with bruxism had greater occlusal imbalances [50].

Another study, conducted by Gümüş et al. in 2013, using the same method of occlusal analysis as in the present study, on the same number of participants, revealed that there are differences between occlusion relationships in participants with bruxism compared to those without bruxism [51].

The present study showed an asymmetric distribution of occlusal forces in the MI position, predominantly on the right hemiarch, especially in the participants with bruxism. The asymmetric distribution of occlusal forces has been associated with the presence of non-working interferences in protrusion movement, which has also been associated with bruxism.

However, the asymmetric distribution of the occlusal forces at the level of the two hemiarches is considered normal by some specialists, being determined by the masticatory type [52,53].

Regarding the evaluation of mandibular movements, the study revealed that in addition to non-working occlusal interferences from protrusion that were associated with bruxism, non-working interferences from laterotrusion movements were associated with increased bruxism indices, specifically for SB. Only the antero-lateral guidance in the left laterotrusion movement was associated with AB.

We have not found in the specialized literature T-Scan studies the association between non-working interferences and bruxism, but over time, this association has been made, in general. The role of non-working interferences from protrusion movement in bruxism was pointed out by Ramfjord in 1961 and Safari et al. in 2013 [21,54]. In the specialized literature, there are explanations of how occlusal interferences from mandibular movements influence the contraction of the masticatory muscles.

When the mandible moves in the protrusion motion, horizontal forces are generated that must be supported by the teeth. The front or anterior teeth can best absorb and dissipate these forces [39]. The anterior teeth must provide adequate guidance for the disocclusion of the posterior teeth. Interferences and non-working contacts from the protrusion movement generate unfavorable forces on the dento-maxillary system by their amplitude and direction [50,51]. Currently, anterior and posterior teeth are considered to function differently. The posterior teeth function effectively in accepting the forces applied during the mouth closing movement. They accept these forces well, primarily because their arched position ensures that the forces are directed into their long axes and are thus efficiently dissipated. The anterior teeth, however, are not favorably positioned on the arches to accept high forces. They are normally positioned with a vestibular incisal orientation to the closing direction so that axial loading is almost impossible. The consequence is that, in situations where the lateral teeth are lost, the front teeth move vestibularly.

The anterior teeth, unlike the posterior teeth, are in the proper position to accept the forces of the eccentric movements of the mandible. Therefore, in general, it can be stated that the posterior teeth function most effectively in fixing the mandible during closure, while the anterior teeth function most effectively in guiding the mandible during eccentric movements [39].

In the laterotrusion movement, regardless of the type of guidance, an immediate disocclusion of the teeth on the non-working hemiarch must occur. Occlusal interferences and premature non-working contacts can be destructive to the dento-maxillary system due to the magnitude and direction of forces that can be applied to the articular structures and teeth [39]. Some studies have shown that non-working interferences are perceived by the neuro-muscular system differently than other types of occlusal contacts. Electromyography studies have shown that all contacts with opposing teeth are inhibitory in nature [39]. In other words, the presence of interarch contacts tends to reduce muscle activity by inhibiting proprioceptors and nociceptors in the periodontal ligaments; however, other electromyographic studies have shown that the presence of non-working contacts on the posterior teeth increases muscle activity [55]. Although increased muscle activity can be demonstrated, the reason for its presence is currently not understood.

One of the most significant reasons why non-working interferences should be avoided relates to the effect they can have on the loading and stability of the temporo-mandibular joints (TMJ). When the mandible is displaced laterally, regardless of the type of guidance, the condyle on the balance side can be influenced by interferences or non-working contacts. When there is no interference or non-working contact, the condyle follows a fixed path, with the medial pole in contact with the medial wall of the glenoid fossa, moving downward and forward. If a non-working contact is present and this contact is significant enough to disable the lateral guidance from the working side, a very unstable mandibular relationship is created.

Remaining only the contact on the non-working side, the mandible can be in a situation where the masticatory muscles can be contracted bilaterally, and one of the condyles can be displaced, depending on the specific muscle activity. In this situation, the support point is established on the non-working contact that can cause the dislocation of one of the joints [39]. It should be noted that not all non-working contacts cause a dislocation, but in patients with bruxism, this situation can occur [39].

The study also highlighted an association between the duration of mandibular movements and bruxism indices, this association being logical because the muscle activity in bruxism also requires the mobilization of the mandible. We have not found any studies in the specialized literature that address this aspect.

In opposition to these studies, in 2003, Manfredini et al. [36] stated that there are not enough studies on the association between occlusal factors and bruxism; in 2004, they showed that there is no relationship between self-reported (clinically established) bruxism and occlusal factors in adults [56,57], a fact also supported by Demir A et al., 2004 [58], Gonçalves LPV et al., 2010 [59], and Cheng HJ et al., 2004 [60].

Later in 2012, Manfredini et al. [61] found a weak correlation between occlusal factors and bruxism, so it did not completely rule out this association.

Taking into account this situation and the lack of clear evidence regarding the involvement of occlusion in bruxism, the treatment of bruxism is indicated only when its complications occur [12,35].

Therapeutic guidelines do not recommend occlusal adjustment but recommend occlusal balancing and protection of tooth structures by means of an occlusal mouth guard.

Brocard et al., 2007 [62] recommended that the treatment of bruxism involves several stages: the behavioral stage with the therapy of psychosocial aspects, the pharmacological stage, the reversible and non-invasive dental stage (occlusal mouth guard), and the irreversible dental stage when oral rehabilitation is needed.

Matusz et al., 2022 [63] recommend educational therapy for a patient about harmful habits, biofeedback, muscle relaxation exercises, occlusal splint therapy (occlusal mouth guard), short-term drug therapy, botulinum toxin injections, psychotherapy (stress reduction, lifestyle change, hypnosis), electrical method, etc.

Paesani 2010 [64] showed that occlusal mouth guards reduce EMG activity but do not eliminate bruxism.

Other studies [65,66] have shown that occlusal mouthguards are effective in bruxism by reducing the activity of the masticatory muscles.

Summarizing the results of the study, we can state that dental occlusion is a component of the reflex mechanisms that control mandibular movements and implicit contractions of the masticatory muscles, but a number of other factors must be considered in the management of bruxism. The occlusal relationships and the forces developed by the oro-facial muscles are in a dynamic balance. Changes in occlusal relationships can influence muscle contraction and cause disturbances in the components of the dento-maxillary system.

Based on the results obtained in this study, we can state with certainty that there are occlusal imbalances in the participants with bruxism, but we cannot say whether these occlusal imbalances maintain the contraction disorders, the contraction disorders cause these occlusal imbalances, or simply the occlusal imbalances coexist with the disorders contraction of the masticatory muscles.

The limits of the study are represented by the number of participants in the study and the fact that occlusal interferences were not determined along the CR—MI pathway, which is also invoked in the etiology of bruxism.

A holistic approach should establish the role of the masticatory system in the etiology of bruxism. Following this, understanding that dysfunctions at the level of the dento-maxillary system (TMD) composed of the dental determinant (dental occlusion), the neuro-muscular determinant (masticatory muscles) and the articular determinant (TMJ) can be taken into account in the etiology of bruxism.

## 5. Conclusions

The study approached a very important and controversial aspect regarding the association between occlusion and bruxism.

The study demonstrated that study participants who had bruxism had a more “unbalanced” occlusion; regarding the distribution of occlusal forces on the two hemiarches, they had increased bruxism indices compared to those without bruxism. The presence of non-working interferences from protrusion movement and group guidance in lateral movements were also associated with increased bruxism indices.

In the current context in which the prevalence of bruxism is increasing, we consider that the approach to bruxism in this study is welcome and requires wider approaches, and the results to materialize in informational and preventive projects, in particular, for high-risk subjects, as well as for specialists involved in bruxism therapy.

Highlighting the risk factors involved in bruxism may be helpful in the management of bruxism.

## Figures and Tables

**Table 1:** Demographic characteristics of the two groups.

	Study Group			Control Group		
	Participants (N/%)	Age Interval	Mean ± SD	Participants (N/%)	Age Interval	Mean ± SD
Females	15 (75%)	20–29	24.2 ± 2.78	15 (75%)	21–53	26.46 ± 7.82
Males	5 (25%)	25–35	29 ± 4.24	5 (25%)	26–47	35 ± 9.11
Overall	20 (100%)	20–35	25.4 ± 3.74	20 (100%)	21–53	28.6 ± 8.76

**Table 2:** The main parameters provided by the dia-BRUXO device.

Parameter		Study Group (n = 20) Mean ± SD	Control Group (n = 20) Mean ± SD
BTI (%)	Awake BTI (ABTI)	2.788 ± 2.381	0.722 ± 0.422
	Sleep BTI (SBTI)	1.055 ± 0.790	0.233 ± 0.201
BWI (%)	Awake BWI (ABWI)	1.178 ± 1.028	0.310 ± 0.214
	Sleep BWI (SBWI)	0.471 ± 0.330	0.098 ± 0.085
BPI (%)	Awake BPI (ABPI)	2.251 ± 1.926	0.585 ± 0.351
	Sleep BPI (SBPI)	0.860 ± 0.633	0.188 ± 0.162

**Table 3:** Distribution of occlusal forces on the hemiarches in the MI position.

	Subgroup (N)	Right Hemiarcade (%)		Left Hemiarcade (%)	
		Mean ± SD	*p* *	Mean ± SD	*p* *
Group	Study (20)	57.080 ± 8.566	0.110	42.920 ± 8.566	0.110
	Control (20)	52.505 ± 7.476		47.425 ± 7.463	
Gender	Females (30)	55.856 ± 7.551	0.187 ^1^	44.110 ± 7.533	0.198 ^1^
	Males (10)	51.600 ± 9.864		48.360 ± 9.854	

* Mann–Whitney U Test. ^1^ Exact significance.

**Table 4:** Duration of occlusion and disocclusion from the MI position by group (seconds).

	Subgroup (N)	Occlusion Duration in MI		Duration of Disocclusion from MI	
		Mean ± SD	*p* *	Mean ± SD	*p* *
Group	Study (20)	1.030 ± 0.671	0.839	0.785 ± 0.444	0.925
	Control (20)	0.968 ± 0.385		0.778 ± 0.418	
Gender	Females (30)	0.999 ± 0.557	0.914 ^1^	0.742 ± 0.407	0.450 ^1^
	Males (10)	1.000 ± 0.519		0.896 ± 0.481	

* Mann–Whitney U Test. ^1^ Exact significance.

**Table 5:** Evaluation of protrusion movement according to the presence of incisal guidance, working and non-working interferences, and premature contacts.

Parameter	Value	Study Group (N = 20)	Control Group (N = 20)	*p* *
Protrusion—the presence of the incisive guidance	Absent	2 (10%)	1 (5%)	0.500
	Present	18 (90%)	19 (95%)	
Protrusion—working side interference	No	10 (50%)	13 (65%)	0.337
	Yes	10 (50%)	7 (35%)	
Protrusion—non-working side interference	No	8 (40%)	13 (65%)	0.113
	Yes	12 (60%)	7 (35%)	
Protrusion—edge-to-edge—premature contacts on the working side	No	14 (70%)	14 (70%)	1
	Yes	6 (30%)	6 (30%)	
Protrusion—edge-to-edge— premature non-working side contacts	No	17 (85%)	14 (70%)	0.225
	Yes	3 (15%)	6 (30%)	

* Chi-Square test.

**Table 6:** Association between bruxism indices and the presence of non-working interferences.

Parameter	Protrusion without Non-Working Interference (N = 21)	Protrusion with Non-Working Interference (N = 19)	*p* *
SBPI (%)	0.378 ± 0.437	0.685 ± 0.660	**0.044** ^1^
SBTI (%)	0.461 ± 0.536	0.845 ± 0.821	**0.044** ^1^
Number of “grinding” events per night	16.760 ± 43.380	28.950 ± 35.442	**0.014** ^1^

* Mann–Whitney U test. ^1^ Statistically significant results.

**Table 7:** Distribution of occlusal forces at the level of the two hemiarches in the MI position, according to the presence of the incisal guidance, the presence of working and non-working occlusal interferences in the protrusion movement.

	Occlusal Status	Distribution of Occlusal Forces on the Right Hemiarch (%)		Distribution of Occlusal Forces on the Left Hemiarch (%)	
		Mean ± SD	*p* *	Mean ± SD	*p* *
Protrusion—incisive guidance	Absent (N = 3)	69.866 ± 0.814	**<0.0005** ^1^	30.133 ± 0.814	**<0.0005** ^1,2^
	Present (N = 37)	53.570 ± 7.316		46.391 ± 7.304	
Protrusion—working interferences	No (N = 23)	55.339 ± 6.621	0.763	44.617 ± 6.597	0.774
	Yes (N = 17)	54.052 ± 10.261		45.923 ± 10.250	
Protrusion—non-working interferences	No (N = 21)	52.333 ± 6.573	**0.027**	47.619 ± 6.569	**0.025** ^2^
	Yes (N = 19)	57.510 ± 9.231		42.468 ± 9.212	

* Mann–Whitney U Test. ^1^ Exact significance. ^2^ Statistically significant results.

**Table 8:** The duration of the protrusion movement according to the presence or absence of interferences (seconds).

Parameter	Group (N)	Protrusion Duration	
		Mean ± SD	*p* *
Protrusion—working interferences	No (23)	2.39 ± 0.945	0.978 *
	Yes (17)	2.36 ± 0.805	
Protrusion—non-working interferences	No (21)	2.31 ± 0.845	0.357
	Yes (19)	2.45 ± 0.929	

* Mann–Whitney U test.

**Table 9:** Duration of protrusion movement according to associations between working and non-working interferences.

Parameter	Group A	Group B	Group C	Group D	*p* *
The duration of the protrusion movement	2.27 ± 0.953	**2.62** ^1^ ± 0.947	2.40 ± 0.547	2.33 ± 0.942	0.651
SBTI	0.48 ± 0.591	0.70 ± 0.448	0.40 ± 0.404	**0.95** ^1^ ± 1.023	0.253
ABTI	1.46 ± 1.407	**2.67** ^1^ ± 3.598	1.53 ± 1.270	1.59 ± 1.369	0.997
SBPI	0.39 ± 0.481	0.57 ± 0.367	0.33 ± 0.332	**0.76** ^1^ ± 0.819	0.253
ABPI	1.18 ± 1.132	**2.15** ^1^ ± 2.906	1.21 ± 0.995	1.30 ± 1.139	0.997

* Kruskal–Wallis test. ^1^ Highest duration

**Table 10:** Evaluation of the left laterotrusion movement according to the type of guidance, the presence of working and non-working interferences, and premature contacts.

Parameter	Guidance Type, The Presence of Interferences and Premature Contacts	Study Group (N = 20)	Control Group (N = 20)	*p* *
Left laterotrusion movement, guidance type	Canine	10 (50%)	18 (90%)	**0.018** ^1^
	Antero-lateral group	8 (40%)	1 (5%)	
	Side group	2 (10%)	1 (5%)	
Left laterotrusion movement, working side interferences	No	4 (20%)	8 (40%)	0.168
	Yes	16 (80%)	12 (60%)	
Left laterotrusion movement, non-working side interferences	No	5 (25%)	10 (50%)	0.102
	Yes	15 (75%)	10 (50%)	
Left laterotrusion movement, edge–edge position, premature working side contacts	No	14 (70%)	13 (65%)	0.736
	Yes	6 (30%)	7 (35%)	
Left laterotrusion movement, edge–edge position, premature non-working side contacts	No	13 (65%)	18 (90%)	0.064
	Yes	7 (35%)	2 (10%)	

* Chi-Square test. ^1^ Statistically significant results.

**Table 11:** The situation of the correlations between the type of guidance in the left laterotrusion movement and the indices of bruxism.

Parameter	Canine Guidance (N = 28)	Antero-Lateral Group Guidance (N = 9)	Side Group Guidance (N = 3)	*p* *
ABPI	1.043 ± 1.095	2.702 ± 2.439	1.057 ± 0.835	**0.030** ^1^
Sleep duration	30.657 ± 8.198	39.255 ± 6.172	31.266 ± 6.296	**0.023** ^1^
Duration of wakefulness	68.917 ± 8.631	60.744 ± 6.172	68.7333 ± 6.296	**0.033** ^1^
The number of nighttime clenching events	16.610 ± 13.356	45.440 ± 31.285	34.670 ± 24.007	**0.015** ^1^
ABTI	1.286 ± 1.348	3.360 ± 3.014	1.310 ± 1.013	**0.027** ^1^

* Kruskal–Wallis H test. ^1^ Statistically significant results.

**Table 12:** Evaluation of the right laterotrusion movement according to the type of guidance, the presence of working and non-working interferences, and premature contacts.

Parameter	Guidance Type, The Presence of Interferences and Premature Contacts	Study Group (N = 20)	Control Group (N = 20)	*p* *
Right laterotrusion movement, guidance type.	Canine	12 (60%)	15 (75%)	0.587
	Antero-lateral group	6 (30%)	4 (20%)	
	Side group	2 (10%)	1 (5%)	
Right side movement, working side interferences.	No	8 (40%)	7 (35%)	0.744
	Yes	12 (60%)	13 (65%)	
Right laterotrusion movement, non-working side interferences.	No	5 (25%)	10 (50%)	0.102
	Yes	15 (75%)	10 (50%)	
Right laterotrusion movement, edge–edge position—premature contacts on the working side	No	13 (65%)	14 (70%)	0.736
	Yes	7 (35%)	6 (30%)	
Right laterotrusion movement, edge–edge position—premature non-working side contacts	No	12 (60%)	14 (70%)	0.507
	Yes	8 (40%)	6 (30%)	

* Chi-Square test.

**Table 13:** Duration of mandibular movements in the two groups of participants (seconds).

Parameter	Study Group (N = 20)	Control Group (N = 20)	*p* *
Duration of protrusion	2.60 ± 0.98	2.16 ± 0.71	0.064
Duration of right laterotrusion	2.81 ± 0.80	2.26 ± 0.78	0.036
Duration of left laterotrusion	2.57 ± 0.87	2.21 ± 0.92	0.110

* Mann–Whitney U test.

**Table 14:** Correlations between duration of mandibular movements and bruxism time indices.

Parameter	Duration (Value/*p* *)				
	Occlusion MI	Disocclusion MI	Protrusion	Left Laterotrusion	Right Laterotrusion
SBTI	−0.046/0.675	−0.054/0.624	0.310/**0.005**	0.305/**0.006** ^1^	0.325/**0.003** ^1^
ABTI	0.018/0.870	−0.023/0.834	0.095/0.388	0.113/0.305	0.162/0.142

* Kendall’s tau-b test. ^1^ Statistically significant results

## Data Availability

The authors declare that the data of this research are available from the corresponding authors upon reasonable request.
